# Supplementary material for: Brain tumour genetic network signatures of survival
Source: Brain. 2023 Sep 4;146(11):4736–54. doi: 10.1093/brain/awad199 (PMC10629773; doi:10.1093/brain/awad199)
Supplement: awad199_Supplementary_Data [file awad199_supplementary_data.zip › brain-2023-00355-File010.pdf]

Brain tumour genetic network signatures of survival

Supplementary material

James K. Ruffle FRCR MSc[1], Samia Mohinta MSc[1], Guilherme Pombo MSc[1], Robert Gray PhD[1], Valeriya Kopanitsa MBBS BSc[1], Faith Lee BSc[1], Sebastian Brandner MD FRCPath[2], Harpreet Hyare FRCR PhD[1], and Parashkev Nachev FRCP PhD[1]

[1]Queen Square Institute of Neurology, University College London, London WC1N 3BG, UK
[2]Division of Neuropathology and Department of Neurodegenerative Disease, Queen Square Institute of Neurology, University College London, London WC1N 3BG, UK

Running title:
Brain tumour genetic network signatures of survival

Correspondence to:
Dr James K Ruffle
Email: j.ruffle@ucl.ac.uk
Address: Institute of Neurology, UCL, London WC1N 3BG, UK

Correspondence may also be addressed to:
Professor Parashkev Nachev
Email: p.nachev@ucl.ac.uk
Address: Institute of Neurology, UCL, London WC1N 3BG, UK

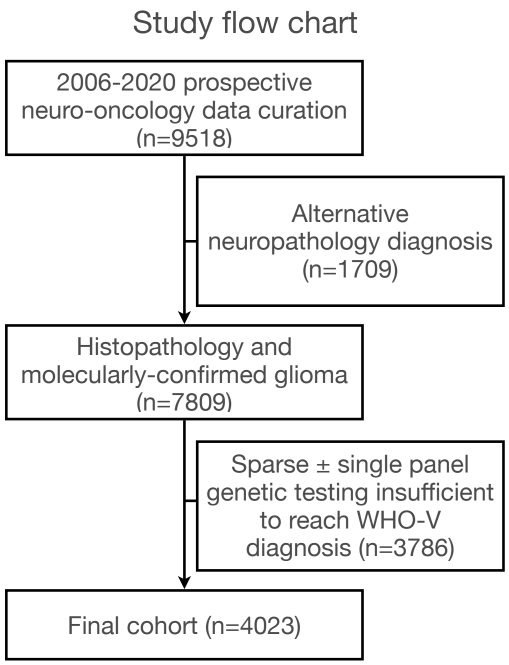

Supplementary Figure 1 - Study flow chart.

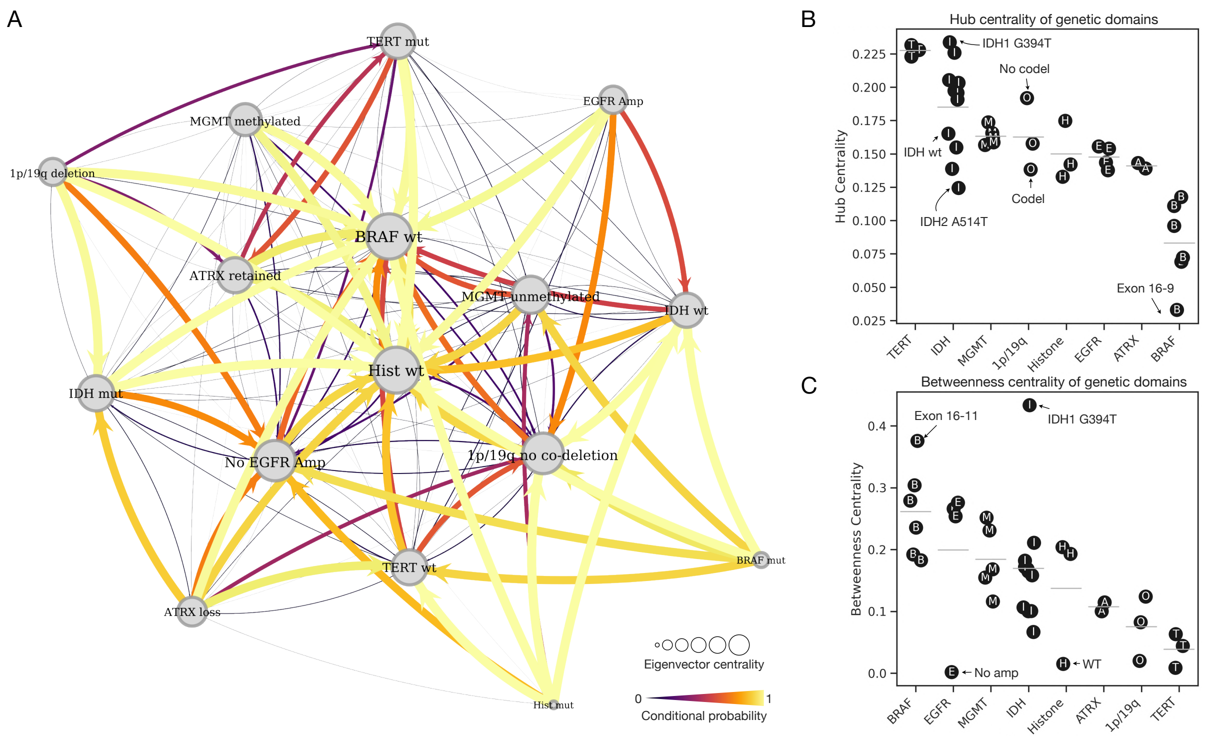

Supplementary Figure 2– Graph models of course tumour genetic domains. A) Spring-block layout of course genetic features - i.e., mutant or wild-type - where the size and colour of edges are proportional to the directional conditional probability, and node size is proportional to the weighted eigenvector centrality. B) There is a significant difference in weighted hub-centrality and betweenness centrality © of tumour genetic factors when organized by genetic domain (both p<0.0001). In panels B-C) points are labelled by their corresponding abbreviation below and outliers are also annotated: A, ATRX; B, BRAF, E, EGFR; H, Histone; I, IDH; M, MGMT, O, 1p/19q; T, TERT.

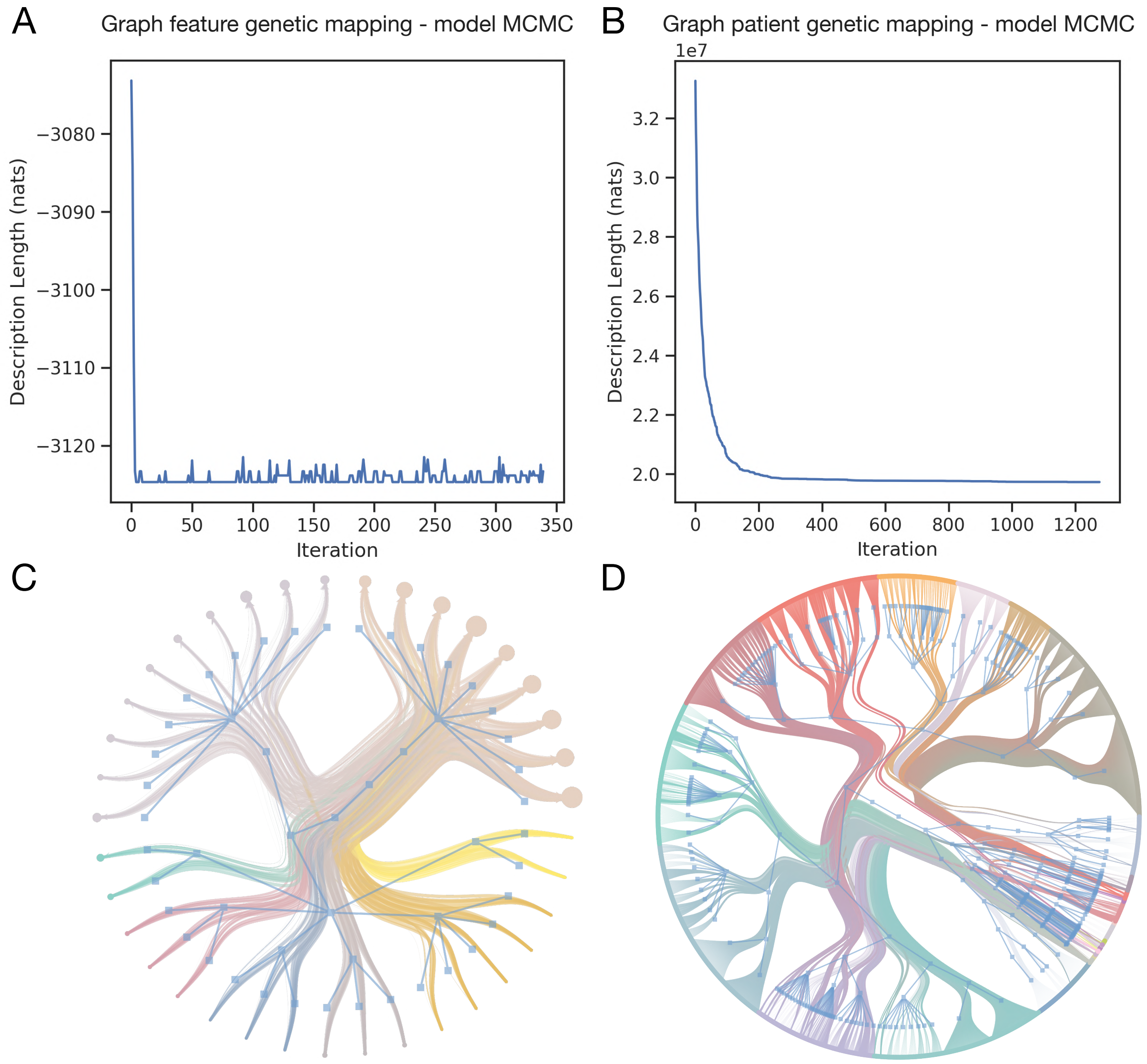

Supplementary Figure 3 – Evidence of model convergence. Iterative reduction in the description length of A) the layered graph feature genetic mapping nested stochastic block model and B) the graph patient genetic mapping model, with MCMC refinement. C) Radial graph result of graph feature genetic mapping from panel A. D) Radial graph result of graph patient genetic mapping from panel B.

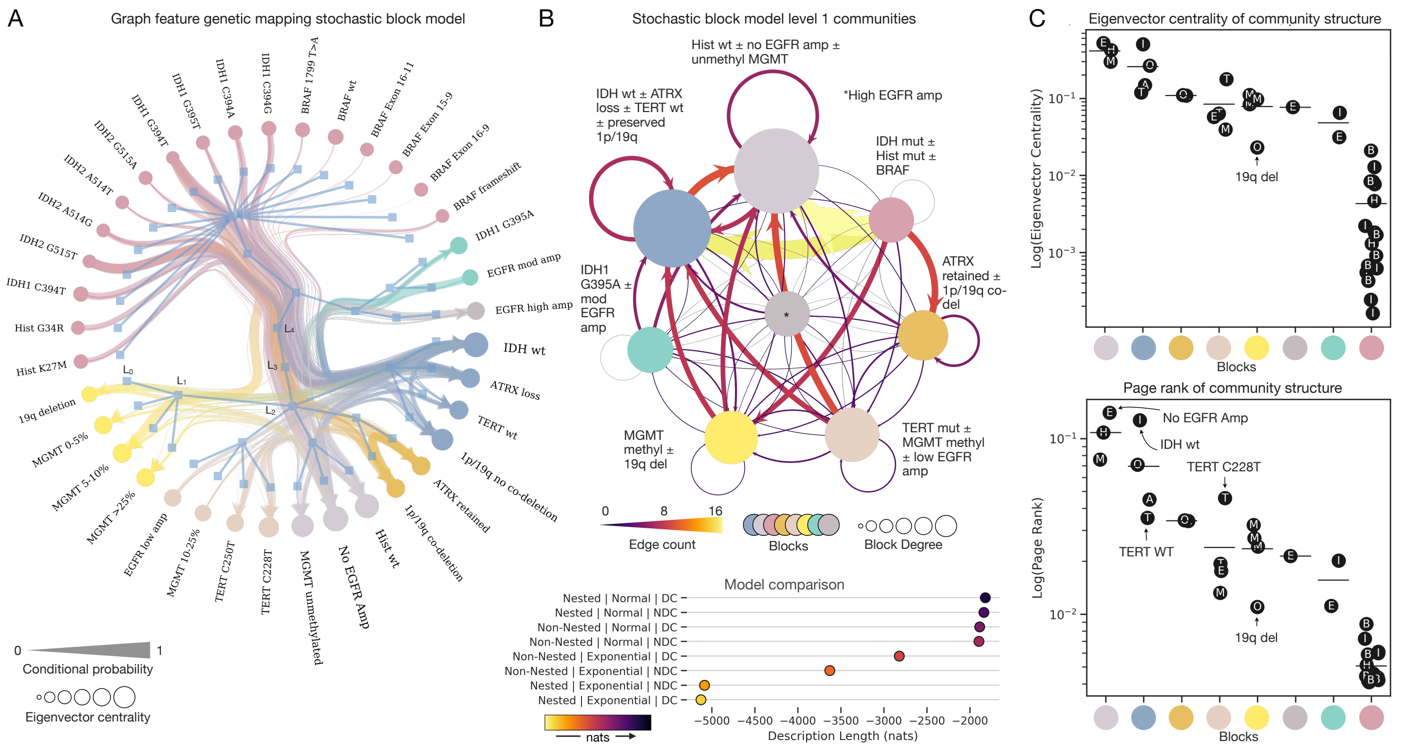

Supplementary Figure 4 – Graph feature genetic mapping identifies systematic brain tumour genetic links. A) Radial graph of layered, nested, degree-corrected, and exponentially weighted stochastic block model revealing the community structure of tumour genetics and their influence upon overall network topology. Communities are colour coded by the first level blocks of the hierarchical community structure. Edges are sized according to their conditional probability. Nodes are sized according to their weighted-eigenvector centrality. Hierarchical levels are annotated from level 0 (L_0) to level 4 (L_4). B) Visualization of the first level hierarchy (L_1) with node colour as per that of panel A. Description lengths for all possible model fits are shown below, illustrating the nested, exponential, and degree corrected (DC) fit to be most suitable. Edge size and colour is proportional to the incidence of edges linking mutations between a given block. Node size is proportional to the degree of the corresponding block. C) There is a significant difference in weighted eigenvector centrality and page rank of tumour genetic factors when organized by stochastic block model community (both $p<0.0001$). In panel C, block colour as per that of panel A, points are labelled by their corresponding abbreviation below and outliers within given communities are also annotated: A, ATRX; B, BRAF, E, EGFR; H, Histone; I, IDH; M, MGMT, O, 1p/19q; T, TERT.

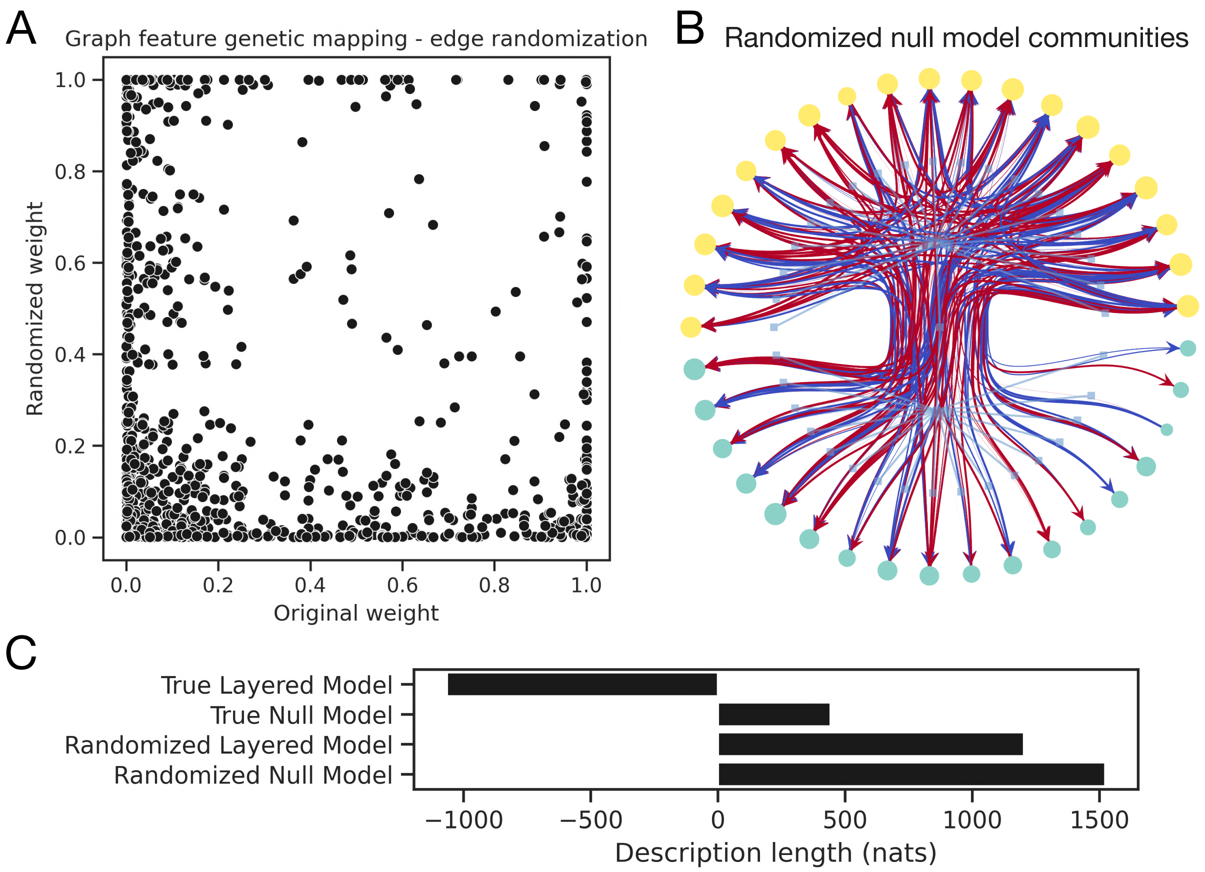

Supplementary Figure 5 – Randomized null models of graph feature genetic mapping. A) Scatterplot depicting edge randomization with original (true) edge weights along the x-axis, and those randomized along the y-axis. B) The community structure from edge-randomized models shows no biologically meaningful (or plausible) segregation. Red edges depict randomized conditional probability edges, and blue edges from randomized sampling frequency. Since the randomized community structure is nonsensical, node labels have been withheld. C) Both randomized layered and null-layered stochastic block models yield larger description lengths than true layered models, indicative of a poor fit.

SEE HTML FILE

Download and open in web browser

Supplementary Figure 6 - Interactive tumour genetic network. Red edges indicate links between loci by conditional probability over and above sampling frequency effects. Blue edges indicate links between loci largely represented by sampling frequency. For conciseness, only the top 25% of edges are shown. The tab icon in top right of screen allows node labels, edge labels, edge, and node sizes to be modified based upon the variety of parameters fitted. Images can also be exported as static images.

SEE HTML FILE

Download and open in web browser

Supplementary Figure 7 - Unthresholded interactive tumour genetic network. Red edges indicate links between loci by conditional probability over and above sampling frequency effects. Blue edges indicate links between loci largely represented by sampling frequency. For a thresholded version of this network, we recommend review of static supplementary figure 2, or interactive supplementary figure 6. The tab icon in top right of screen allows node labels, edge labels, edge, and node sizes to be modified based upon the variety of parameters fitted. Images can also be exported as static images.

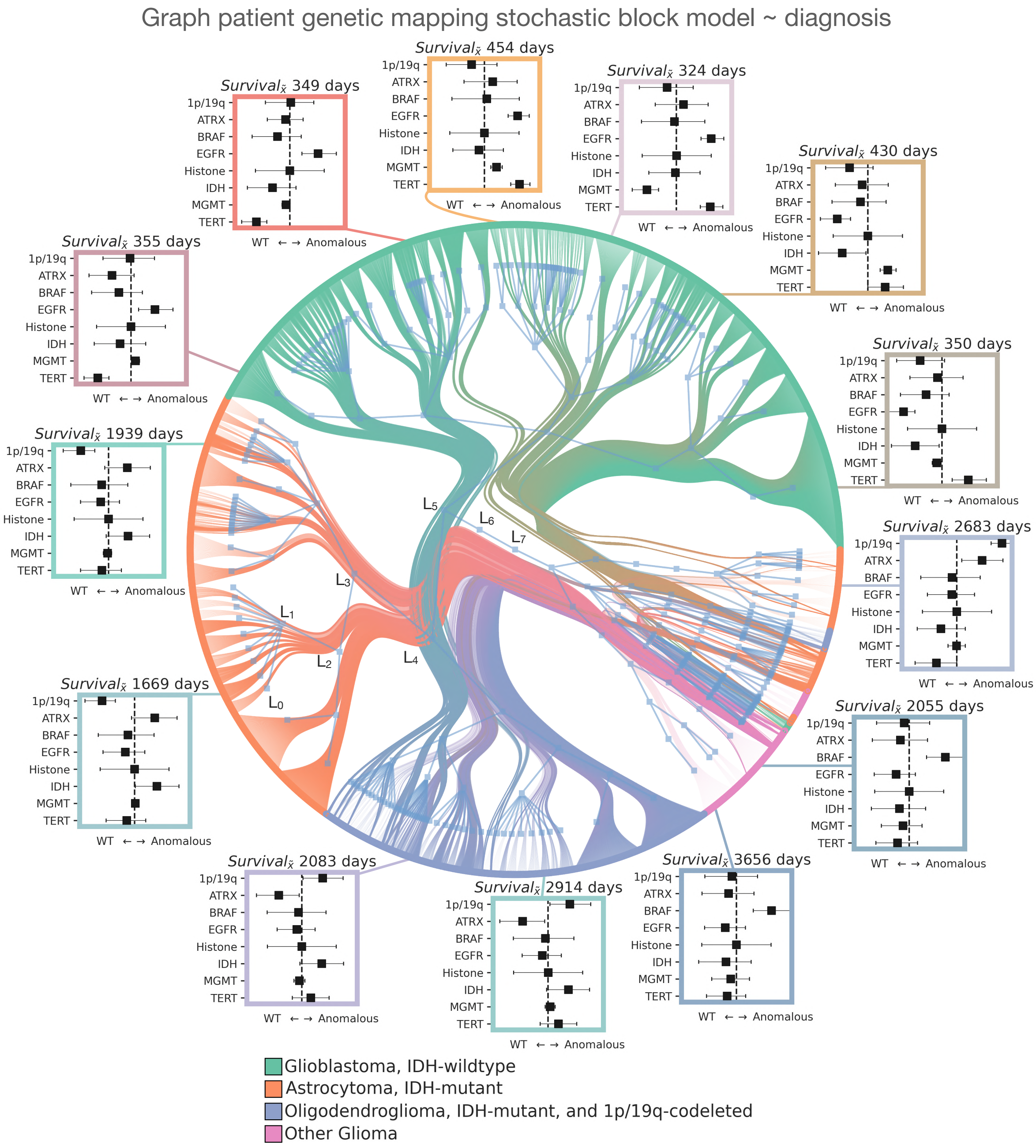

Supplementary Figure 8 – Graph patient genetic mapping recovers overall diagnosis. Radial graph of nested, degree-corrected, and multivariate binomially weighted stochastic block model revealing the community structure of patients and the genetics of their brain tumour. Patient nodes are color-coded by diagnosis, as per the colour-key. Around the radial graph are the breakdown of median survival and box and whisker plots for the coefficients and 95% confidence intervals of genetic loadings, where the coloured border of the plots depicts the corresponding community. All boxplots wherein the error-bar does not cross the vertical zero-line are significant, with features left of the vertical zero-line favouring the wild-type, and right of the zero-line favouring mutation. Note only the minimum spanning tree of the graph is shown, owing to visualisation constraints.

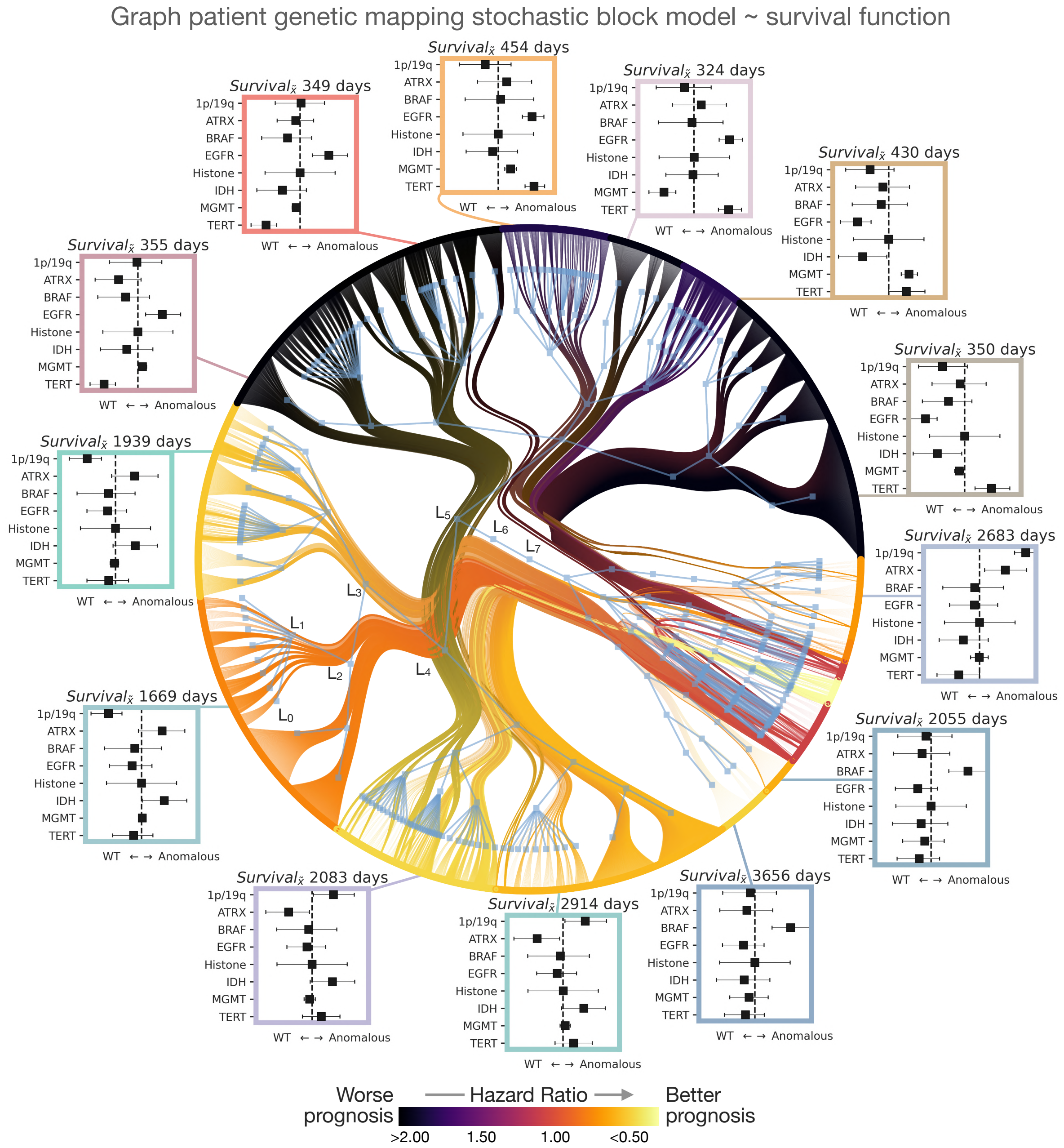

Supplementary Figure 9 – Graph patient genetic mapping recovers offers more personalised survival predictions. Radial graph of nested, degree-corrected, and multivariate binomially weighted stochastic block model revealing the community structure of patients and the genetics of their brain tumour. Patient nodes are color-coded by the hazard ratio of Cox's proportional hazard model. Around the radial graph are the breakdown of median survival and box and whisker plots for the coefficients and 95% confidence intervals of genetic loadings, where the coloured border of the plots depicts the corresponding community. All boxplots wherein the error-bar does not cross the vertical zero-line are significant, with features left of the vertical zero-line favouring the wild-type, and right of the zero-line favouring mutation. Note only the minimum spanning tree of the graph is shown, owing to visualisation constraints.

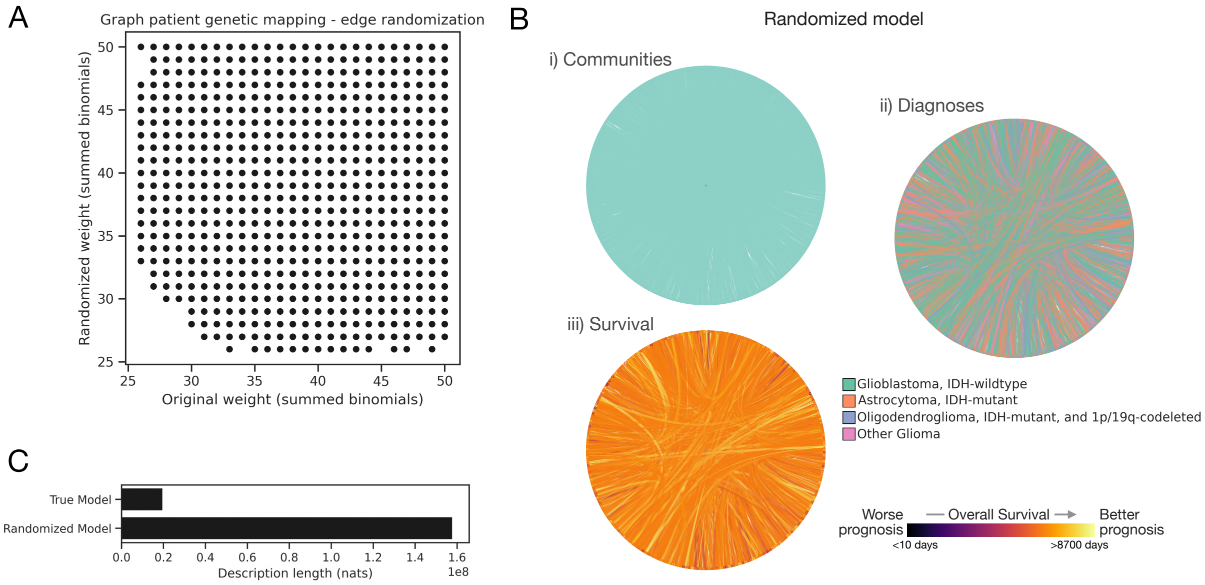

Supplementary Figure 10 – Randomized null models of graph patient genetic mapping. A) Scatterplot depicting edge randomisation with the sum of the original (true) edge weights along the x-axis, and the sum of those randomized along the y-axis. B) There is no community structure from the randomized null model (panel i), bearing no relation to either diagnosis (panel ii), or mean survival (panel iii). C) The randomized stochastic block model yields a far larger description lengths than the true model, indicative of a poor fit.

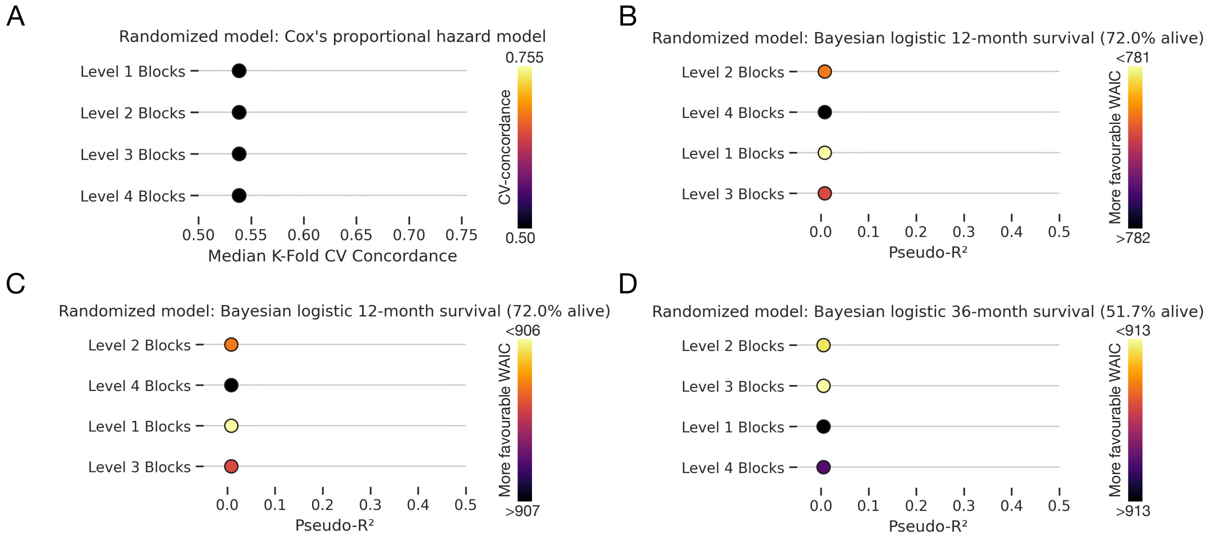

Supplementary Figure 11 – Randomized null survival models. A) Cox's proportional hazard, B) Bayesian logistic regression for 12-month, C) 24-month, and D) 36-month survival using the results of the randomized baseline model (Supplementary Figure 10) yields no predictive power in survival forecasting.
