# Supplementary material for: Brain tumour genetic network signatures of survival
Source: Brain. 2023 Sep 4;146(11):4736–54. doi: 10.1093/brain/awad199 (PMC10629773; doi:10.1093/brain/awad199)
Supplement: awad199_Supplementary_Data [file awad199_supplementary_data.zip › brain-2023-00355-File011.html]

Details for selected element

General

App state

Reset

Display mode

Enter full screen

Export

SVG
PNG
JPG

Data selection

Graph

Node label text

Edge label text

Node size

Normalize

Minimum

Maximum

Edge size

Normalize

Minimum

Maximum

Nodes

Visibility

Show nodes

Size

Scaling factor

Position

Release fixed nodes

Drag behavior

Fix node position

Hover behavior

Show neighborhood

Show tooltips (if provided)

Node images

Visibility

Show node images

Size

Scaling factor

Node labels

Visibility

Show node labels

Show borders

Size

Scaling factor

Rotation

Angle

Edges

Visibility

Show edges

Size

Scaling factor

Form

Curvature

Hover behavior

Show tooltips (if provided)

Edge labels

Visibility

Show edge labels

Show borders

Size

Scaling factor

Rotation

Angle

Layout algorithm

Simulation

Active

Many-body force

On

Strength

Theta

Use minimum distance

Min

Use maximum distance

Max

Links force

On

Distance

Strength

Collision force

On

Radius

Strength

x-positioning force

On

Strength

y-positioning force

On

Strength

Centering force

On
